# Reorienting adolescent sexual and reproductive health research: reflections from an international conference

**DOI:** 10.1186/s12978-016-0117-0

**Published:** 2016-01-13

**Authors:** Kristien Michielsen, Sara De Meyer, Olena Ivanova, Ragnar Anderson, Peter Decat, Céline Herbiet, Caroline W. Kabiru, Evert Ketting, James Lees, Caroline Moreau, Deborah L. Tolman, Ine Vanwesenbeeck, Bernardo Vega, Elizabeth Verhetsel, Venkatraman Chandra-Mouli

**Affiliations:** 1 International Centre for Reproductive Health, Ghent University, Ghent, Belgium; 2 Guttmacher Institute, New York, USA; 3 Department of Family Medicine and Primary Health Care, Ghent University, Ghent, Belgium; 4 Butterfly Works, Amsterdam, The Netherlands; 5 African Population and Health Research Center, Nairobi, Kenya; 6 Radboud University, Nijmegen, The Netherlands; 7 University of the Western Cape, Cape Town, South Africa; 8 Johns Hopkins Bloomberg School of Public Health, Baltimore, USA; 9 Hunter College and The Graduate Center, CUNY, New York, USA; 10 Rutgers & Utrecht University, Utrecht, The Netherlands; 11 University of Cuenca, Cuenca, Ecuador; 12 Sensoa, Antwerp, Belgium; 13 Department of Reproductive Health and Research, World Health Organization, Geneva, Switzerland

**Keywords:** Adolescents, Sexual and reproductive health, Evaluation research, Social determinants

## Abstract

On December 4th 2014, the International Centre for Reproductive Health (ICRH) at Ghent University organized an international conference on adolescent sexual and reproductive health (ASRH) and well-being. This viewpoint highlights two key messages of the conference - 1) ASRH promotion is broadening on different levels and 2) this broadening has important implications for research and interventions – that can guide this research field into the next decade. Adolescent sexuality has long been equated with risk and danger. However, throughout the presentations, it became clear that ASRH and related promotion efforts are broadening on different levels: from risk to well-being, from targeted and individual to comprehensive and structural, from knowledge transfer to innovative tools. However, indicators to measure adolescent sexuality that should accompany this broadening trend, are lacking. While public health related indicators (HIV/STIs, pregnancies) and their behavioral proxies (e.g. condom use, number of partners) are well developed and documented, there is a lack of consensus on indicators for the broader construct of adolescent sexuality, including sexual well-being and aspects of positive sexuality. Furthermore, the debate during the conference clearly indicated that experimental designs may not be the only appropriate study design to measure effectiveness of comprehensive, context-specific and long-term ASRH programmes, and that alternatives need to be identified and applied. Presenters at the conference clearly expressed the need to develop validated tools to measure different sub-constructs of adolescent sexuality and environmental factors. There was a plea to combine (quasi-)experimental effectiveness studies with evaluations of the development and implementation of ASRH promotion initiatives.

## Background

On December 4^th^ 2014, the International Centre for Reproductive Health (ICRH) at Ghent University organized an international conference on “Adolescent Sexual and Reproductive Health and Well-being: How to align the need for complex interventions with the call for more evidence?” in Ghent, Belgium. The conference gathered over a hundred participants with a large variety of nationalities and backgrounds: researchers, advocates, implementers, students, and policy makers. High profile speakers reflected upon how the ASRH field has evolved and discussed possible future developments in adolescent sexual and reproductive health (ASRH) research. The conference covered three main topics: 1) innovations in ASRH promotion; 2) understanding, conceptualizing and measuring the concept of adolescent sexual and reproductive well-being; 3) generating evidence of the effectiveness of comprehensive ASRH promotion programmes. Topics 1 and 2 were introduced in the form of presentations, while topic 3 was addressed in a debate.

An overview presentation by Venkatraman Chandra-Mouli of the World Health Organization on ASRH in the past two decades, highlighted the limited and patchy progress in ASRH [1]: the number of births to girls aged 15–19 years declined globally from 64 in 1990 to 54 in 2011 (per 1000 girls), however the birth rate in sub-Saharan Africa remained high at 117 [2]; progress in HIV prevention was uneven across different world regions; for example, while new infections decreased substantially in Eastern and Southern Africa, they remained stable in Asia and the Pacific [2]; nearly 30 % of adolescent girls (15–19 years) have experienced intimate partner violence [3]; and reductions in child marriages are limited [4]. But he and other speakers stressed that even with continuing challenges, much progress had been made in research, implementation and advocacy over the past two decades, and that the way forward is clear. The studies and projects presented at the conference provided new insights into the status of ASRH research and suggested priorities on how to move forward.

This viewpoint highlights two key messages of the conference: 1) ASRH promotion is broadening on different levels (content, target group, tools), and 2) this broadening has important implications for research and interventions.

## Review

### Adolescent sexual and reproductive health promotion is broadening on different levels

#### Dominant ASRH promotion

Partly because of its serious health consequences, adolescent sexuality has long been equated with risk and danger [5]. This is reflected in research and interventions on the topic. Deborah Tolman of Hunter College and The Graduate Center, CUNY (USA) presented a review of literature on adolescent sexuality (2000–2009) in which 80 % of the identified 732 studies dealt with risk, risk prevention, or identifying predictors of negative outcomes [6]. In particular, in low and middle-income regions, where negative consequences of sex are most tangible, research on ASRH is risk oriented [7]. This risk orientation is reflected in researchers’ choices of research questions that aim to understand the determinants of sexual risk-taking (e.g. unprotected sexual intercourse), focus on negative consequences of sexual behavior (e.g. HIV/STI) and how to prevent them (e.g. abstinence) [8]. Interventions based on this risk paradigm mostly target a specific health issue or behavior on the individual level.

However, throughout the presentations, it became clear that ASRH and its promotion efforts are broadening on different levels: from risk to well-being, from targeted and individual to comprehensive and structural, from knowledge transfer to innovative tools.

#### From risk to well-being

The past decade witnessed the emergence of a small but critical mass of studies accepting adolescent sexuality, particularly for girls, as a normal and expected aspect of human development. Tolman indicated that in 20 % of the studies in the previously mentioned literature review, sexual development of adolescents was considered normal. These studies, largely coming from the USA and the UK, go beyond risks, and integrate “positive” dimensions with risk management dimensions. The focus shifts from preventing problems to learning about adolescents’ sexual selves and bodies, intimate partners and relationships, various perspectives on community and cultural values and sexual rights [6]. Taking this point further, Ragnar Anderson from the Guttmacher Institute presented an increasing body of research on the importance of sexual pleasure for sexual well-being, though this research is still very Western focused and limited to adults [9].

#### From targeted and individual to comprehensive and structural

The conference also focused on comprehensive approaches. Evert Ketting of Radboud University (Netherlands) presented the concept of Holistic Sexuality Education (HSE), based on the European Standards for Sexuality Education. Holistic Sexuality Education is not an intervention, but a learning process, that starts from a holistic concept of (sexual) well-being, and goes beyond public health. It is a long-term process, involving many stakeholders, starting early in life and is spread out in an age- and developmentally appropriate way throughout childhood and adolescence. It does not try to change sexual behavior, rather it wants to enable people to, ultimately, achieve a safe and satisfactory sexual life [8, 10]. Similarly, the Adolescent Girls Initiative in Kenya, presented by Caroline Kabiru of the African Population and Health Research Center (Kenya), aims to improve sexual well-being, by focusing on interventions in four sectors—health, wealth creation, education and prevention of violence. The rationale is that ASRH can only be sustainably improved if these other areas are addressed. Different combinations of interventions are tested to determine the most cost-effective in improving girls’ well-being.

In his presentation, James Lees of the University of the Western Cape (South Africa) presented a structural approach to ASRH, tackling ASRH on a social level by targeting prejudices of teachers. He talked about his experiences in developing and teaching the first accredited teacher-training course on sexual diversity in Africa. The course was developed at the request of students, who, during teaching practice sessions in schools, saw increased questioning of, and bullying related to, sexual diversity by students. The course created a safe space for future teachers to confront their own homophobic beliefs and attitudes, allowing them to discover their own roles and commitment as teachers to defend the rights of their colleagues and future pupils to be lesbian, gay, bisexual, transgender or intersex. By working to help teachers align their teaching practice with Section Nine of the South African Constitution that guarantees rights and protections to all people regardless of sexual orientation, the course has the potential to reach a large number of children and adolescents and could have tremendous impact on ASRH.

The increased comprehensiveness of ASRH promotion is not limited to content only, but extends to the inclusion of key stakeholders. While this is often seen as an obligation, Elizabeth Verhetsel from the Flemish Expertise Centre for Sexual Health, Sensoa, illustrated the added value of meaningful youth involvement in their activities and campaigns. She walked the audience through their experiences from the early days (i.e., obligation to have one adolescent on their board) to a structure where adolescents are truly embedded in all aspects of their activities, a demanding but highly positive experience.

#### From knowledge transfer to innovative tools

Other important contributions were made by Bernardo Vega of Cuenca University in Ecuador and Peter Decat of ICRH. In the Latin American context in which they are working, access to contraceptives is difficult for adolescents. Even though contraceptives are available in pharmacies and even free of charge from public clinics, judgmental attitudes of health workers and pharmacists hinder young people’s contraceptive use, contributing to high rates of teenage pregnancies. Decat suggested giving adolescents direct access to contraceptives through an automatic distributer to be used with a personalized card. M-health is often discussed in the context of reaching adolescents with health messages. In addition to this, based on his work in Ecuador, Vega stressed the importance of using information technologies (helpline, sms services) to give adolescents a direct and anonymous line to youth-friendly health providers. Based on his work, Vega also indicated the use of social media, not just for reaching adolescents but to mobilize policy makers. While the latter would not respond to official letters and paper invitations, a public Tweet or Facebook message guaranteed an immediate reaction and mobilization. Also Butterfly Works, a social innovation studio, empowers youth and organizations to use relevant technology and design in addressing the SRHR needs of adolescents. Céline Herbiet presented ‘Oh my body’, a new project providing young people with contextualised and youth friendly information about SRHR on their mobile devices.

### Broadening of ASRH promotion has implications for research

#### The need for (better) indicators

As the concept of adolescent sexuality and its related promotion is broadening, research efforts need to evolve in the same direction. However, indicators to measure adolescent sexuality and its different sub-constructs are lacking. While public health related indicators (HIV/STIs, pregnancies) and their behavioral proxies (e.g. condom use, number of partners, age at sexual debut) are well developed and documented, there is a lack of consensus on indicators for the broader construct of adolescent sexuality, including sexual well-being indicators and aspects of positive sexuality among adolescents, such as body pride, self-esteem and positive sexual experiences, even more so for early adolescents. While these indicators are important in their own right, they are also essential in order to study their association with and impact on the more narrowly defined sexual health indicators. Presenters at the conference clearly expressed the need to develop validated tools to measure different sub-constructs of adolescent sexuality and environmental factors.

Caroline Moreau of Johns Hopkins University presented preliminary results of a population-based study on sexual dysfunction among adolescents and young people in France. The data indicate that, in general, most adolescents and youth experience a pleasurable and satisfying sexual life. Nevertheless, youth experience sexual problems that have a gendered distribution, with girls consistently reporting more problems. Moreau equally emphasized the lack of knowledge about the link between sexual dysfunction and SRH outcomes, and the lack of validated measures for sexual dysfunction. In her presentation, Ragnar Anderson complemented Moreau by talking about the opposite of sexual dysfunction, sexual pleasure – defined as physical, emotional, psychological enjoyment from sexual or erotic activities – and its link to sexual and overall health. Based primarily on studies conducted in Western settings, Anderson indicated an established link between sexual pleasure on the one hand and (among others) the selection of a contraceptive method, psychological satisfaction, high self-esteem, sexual assertiveness, stronger sense of social connection, and decreased stress and anxiety on the other. However, most studies focus on adults; thus, the role of sexual pleasure in the general well-being of adolescents is yet unclear. Anderson equally lamented the lack of good indicators to measure sexual pleasure, which are often dichotomous and do not allow nuanced measurements.

Ine Vanwesenbeeck of Rutgers critically reflected upon the broadly used term of sexual empowerment. While sexual empowerment has multiple indivisible degrees (having choice, using choice, achieving choice) and dimensions (psychological, physical, social, economic, legal) it is often used in a narrow sense, focusing only on the individual outcome level. But there are structural aspects to empowerment as well, in terms of resources and opportunities. In addition, empowerment is as much a process as it is an outcome and both processes and outcomes may differ across individuals, contexts, times and places. Due to this complexity, sexual empowerment is often considered too complex to measure. Nevertheless, this complexity should challenge rather than simply frustrate researchers and inspire them to develop a multidimensional and multi-method measure that can be used to study how, when and which dimensions of sexual empowerment can have positive outcomes for whom. The same argument can be made for all sub-constructs of adolescent sexuality.

While these broader conceptions of ASRH should not draw the focus on sexual health outcomes away from HIV, STIs and pregnancy, which are still highly relevant, there was consensus throughout the conference presentations and audience remarks, that investing in the development of good indicators to measure the primarily positive sub-constructs of adolescent sexual well-being rather than solely risk, is urgent. A more comprehensive, ecological approach to measuring young people’s ASRH is crucial to showcasing its benefits, to better understand how these can be improved and how young people’s SRH rights can be guaranteed in order to accelerate the limited and patchy progress in ASRH over the past years.

#### Alternative evaluation designs

All ASRH promotion efforts described in the first section are new and innovative, and their effectiveness has yet to be demonstrated. In order to respond to the call for evidence-based policy making and to learn from and improve our efforts, evidence of (in)effectiveness needs to be studied. However, the debate during the conference clearly indicated that the current focus on experimental designs may not be the only appropriate study design to measure effectiveness of comprehensive, context-specific and long-term ASRH programmes.

There was a plea to combine (quasi-)experimental effectiveness studies with evaluations of the development and implementation of ASRH promotion initiatives. For example, most adolescents and youth still do not yet have access to comprehensive or holistic sexuality education (C/HSE), despite repeated intergovernmental agreements to provide it, support from the UN system, and considerable project-level experience in a wide range of countries and research showing its effectiveness [11]; C/HSE programmes reach youth too late, they are delivered with ‘watered down’ content, and they do not reach marginalized young people [12]. In this case, the focus should not only be on the effect and impact of C/HSE on individual behaviors, but to an equal measure on the quality of the content, the barriers and facilitating factors in its implementation and the effect on environmental factors, in order to gain better insights into why certain interventions and programmes work or not in a particular context [8]. Finally, the role of the target audience – i.e. adolescents – in evaluating ASRH promotion efforts cannot be forgotten or overlooked as it so frequently is in evaluation efforts [8].

## Conclusion

Within the community of people working on ASRH present at the conference, there was a common agreement that the field of adolescent sexual and reproductive health is shifting: from risk oriented to well-being, from targeted-individual interventions to comprehensive programmes and from knowledge transfer to innovative tools. The experts jointly accepted the challenge of developing valid measures to study ASRH and well-being, and to develop alternative study designs to measure effectiveness of ASRH promotion programmes. There was a clear call for open collaboration in this process, including researchers, interventionists, policy makers, donors, and not the least, adolescents themselves.

## Abbreviations

ASRH: Adolescent sexual and reproductive health
C/HSE: Comprehensive or Holistic sexuality education
HIV: Human Immunodeficiency Virus
HSE: Holistic Sexuality Education
SRH: Sexual and reproductive health
SRHR: Sexual and reproductive health and rights
STIs: Sexually Transmitted Infections
